# Magnetic Response of Excitons and Excitonic Complexes in Defective Hexyl Ammonium Lead Iodide Self-Assembled Quantum Wells

**DOI:** 10.1021/acsnano.5c07593

**Published:** 2026-03-27

**Authors:** Maria F. Munoz, Destiny Konadu, Adedayo M. Sanni, Casandra L. Ward, Atish Ghosh, Amos Afugu, Zhen-Fei Liu, Angela R. Hight Walker, Aaron S. Rury

**Affiliations:** † Quantum Measurement Division, 96990 National Institute of Standards and Technology, Gaithersburg, Maryland 20899, United States; ‡ Department of Chemistry, 2954 Wayne State University, Detroit, Michigan 48202, United States; § Lumigen Instrument Center, Wayne State University, Detroit, Michigan 48202, United States; ∥ Materials Structural Dynamics Laboratory, Wayne State University, Detroit, Michigan 48202, United States

**Keywords:** hybrid perovskites, defect chemistry, magneto-spectroscopy, exciton−exciton interactions, liquid interfacial chemistry, exciton complexes

## Abstract

Correlating the magnetic behavior of low-dimensional semiconductors with the chemistry used to form these excitonic materials remains crucial to the development of devices applied to quantum technologies. In this study, we apply large magnetic fields during the collection of low-temperature photoluminescence (PL) to assess the magnetic properties of excitons in hexyl ammonium lead iodide (HA_2_PbI_4_) self-assembled quantum wells (SAQWs) formed at liquid–liquid interfaces. The effect of incident laser power and temperature was used to assign lower energy features in these samples’ PL spectra to both excitons trapped at defect sites and trions. Measured peaks shifts allow us to estimate coupling between the applied magnetic fields and the charged defect excitons. Our conclusions are supported by density functional theory calculations on supercells of the proposed defective HA_2_PbI_4_ SAQW structure. Additionally, we find an anomalous magnetic response from trion states that resembles the behavior of interacting excitonic complexes in similar, quantum-confined materials at low temperatures. These results highlight the crucial role that chemical conditions can play in the magnetic response of 2D semiconductors applied in quantum technologies.

## Graphical abstract

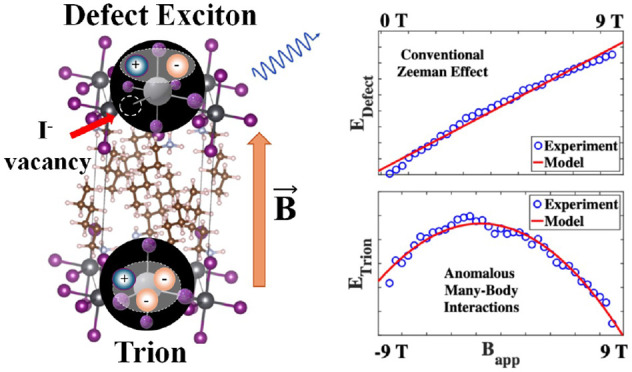

## Introduction

The production of single photons from solid-state material systems remains crucial for the development of light-based quantum information processing and computing technologies.^1–4^ Production of single photons in the visible region of the electromagnetic spectrum provides the means not only leverage the quantum nature of light particles, but also introduce additional quantum correlations via parametric downconversion.^5^ When using visible photons, this nonlinear optical process can produce near-IR photon pairs whose energies, momenta, and polarization can be correlated in nonclassical ways.^6^ Researchers’ ability to form these non-classically correlated photon pairs in the near-IR could introduce novel means to transport quantum information and quantum key distribution via existing fiber optic infrastructure.^6^

Given the need to extract single photons with high efficiencies, point defects of insulators and semiconductors have emerged as important systems to study.^7–9^ For example, the combined N impurity-C vacancy sites in diamond produce electronic states inside the material’s bandgap whose energies can be controlled with external magnetic fields. Given the long electronic coherence of these states due to the rigid lattice of diamond, specific quantum information and computing protocols have been demonstrated using these states.^7,10–15^ However, the chemical control of NV centers in diamond remains challenging, which suggests that other material platforms must be considered.

Excitons represent important excitations in condensed matter systems, since they absorb and emit light readily. While light emission from excitons remains an important fundamental topic for the materials science research community, luminescence due to exciton recombination at the defective regions of low-dimensional semiconducting materials has appeared as a promising direction to produce single visible photons on demand. In the context of transition metal dichalcogenides (TMDCs), researchers have demonstrated that defect densities and energies can be controlled chemically and through external forces such as temperature, electric fields, and magnetic fields.^16–19^ Given the significant quantum and dielectric confinement of charge carriers present in low-dimensional semiconductors, excitons are easily trapped at defect sites in these materials. Prototypically, trapped excitons bind more tightly than those excitations that diffuse through the material lattice freely, which produces light emission in the transparent regions of the materials’ optical spectra. When cooled to low temperatures, the emission spectra of excitons trapped at defects in TMDCs appear as narrow features due to the long electronic coherence times associated with their excitation.^16,18^ The application of single photons emitted by these defect centers to quantum information and computing remains an open area of research. Researchers produce defective regions in TMDCs using a variety of techniques, ranging from irradiation with charged particles^20–23^ to intense laser fields.^24,25^ However, these methods produce a variety of defect sites whose roles in photophysical processes remain unclear, which inhibit the development of these material platforms as controllable single-photon emitters.

Hybrid organic–inorganic perovskite-like (HOIP) materials can be formed into pseudo-low-dimensional systems when researchers use appropriately large molecular cation spacers to drive the formation of self-assembled quantum well (SAQW) superlattices and possess bright excitons due to quantum confinement.^26–31^ By adjusting the thickness of inorganic layers in these materials, researchers can change the quantum and dielectric confinement of electronic excitations.^32,33^ These changes to confinement parameters allow one to produce exciton binding energies between tens to hundreds of meV and drive their binding on ultrafast time scales following production of free carriers in dispersive electronic bands.^34^ Furthermore, researchers can modulate the energies of these exciton transitions from the near-UV to the visible to the near-IR by changing the identities of the halide anions used to form the inorganic layers from Cl^–^ to Br^–^ to I^–^, respectively. Moreover, these materials can be processed in solution using earth-abundant chemical constituents.

In the context of optoelectronic applications, most of the interest in the defective regions of HOIP SAQW materials has focused on the appearance of broadband light emission below the energies of excitons free to diffuse through these materials’ lattices.^35–44^ There has been little study of how these defects can trap excitons and lead to narrow features in light emission spectra, which may be useful for single-photon-emitting platforms. Recently, we have demonstrated that defect-induced light emission from HOIP SAQWs can be controlled chemically using materials synthesis at a liquid–liquid interface.^43,44^ Broadband features appear in the light emission spectra of materials formed in the presence of excess acid and molecular amine in the organic layer.^43^ Further studies showed that nearly stoichiometric quantities of organic and inorganic constituents can drive the formation of materials whose light emission spectra possess narrow features at reduced temperatures when using this liquid–liquid interfacial synthesis method.^44^ Despite these intriguing results, it remains unclear how the formation of the materials using interfacial liquid–liquid methods affects important fundamental physical properties, such as the magnetic response of excitons near defects and excitonic complexes. Assessing the suitability of these materials for quantum information processing applications will depend critically on determining their fundamental physical properties.

Magneto-optical studies have been critical to understanding the roles of spin–orbit, crystal field, and exchange coupling in the energetics of bright and dark excitons in HOIP SAQWs.^45–51^ However, it remains unclear how exciton-magnetic interactions should change in the presence of defects or excitonic complexes in these materials. This uncertainty suggests that further studies of the fundamental photophysics of defective HOIP SAQWs are needed to assess their suitability for quantum information sciences (QIS) applications.

In this study, we demonstrate several important features of the low-temperature, magnetic response of a HOIP SAQW system grown at liquid–liquid interfaces. First, we find that these samples possess high-quality surfaces that enable us to resolve narrow features in PL measurements carried out at 10 K without the need for mechanical exfoliation or capping with low-dimensional insulators. Second, we find that more defective samples possess lower energy PL features consistent with excitons affected by defects in these samples. We use computational results to rationalize changes in the magnetic response of trapped excitons with respect to the angular momentum of nearby atomic orbitals, consistent with our chemical approach. Third, we find PL signals consistent with trion excitations, which have not been previously observed in hexyl ammonium lead iodide. Additionally, we establish that trions in our samples respond to applied magnetic fields similarly to interacting excitonic complexes in other low-dimensional semiconductors.^52–54^ These results demonstrate the ability of liquid–liquid methods to control how light emission features in 2D perovskite spectra depend on the chemical conditions under which these materials form and the external forces applied to them. The control of materials made with these methods could be leveraged to make them more suitable for photon-based QIS.

## Results and Discussion

We show the basic principles of our liquid–liquid interfacial synthetic approach in Figure 1(a). Our previous X-ray diffraction studies show hexyl ammonium lead iodide (HA_2_PbI_4_) forms in a monoclinic phase when we use this synthetic method,^43^ which had been established independently.^55^ We show this structure in Figure 1(b). We have reestablished that the material maintains this structure down to 100 K using single-crystal X-ray diffraction measurements, as described in the Methods section below. We report the parameters of the structure refined from those results in Table S1. Previously, we characterized these materials with X-ray photoelectron spectroscopy, Raman spectroscopy, scanning electron microscopy, and powder X-ray diffraction measurements to demonstrate their behavior conforms with the structure reported here and depends on the density of defects we introduce via our interfacial chemical synthesis.^43^

**Figure 1:**
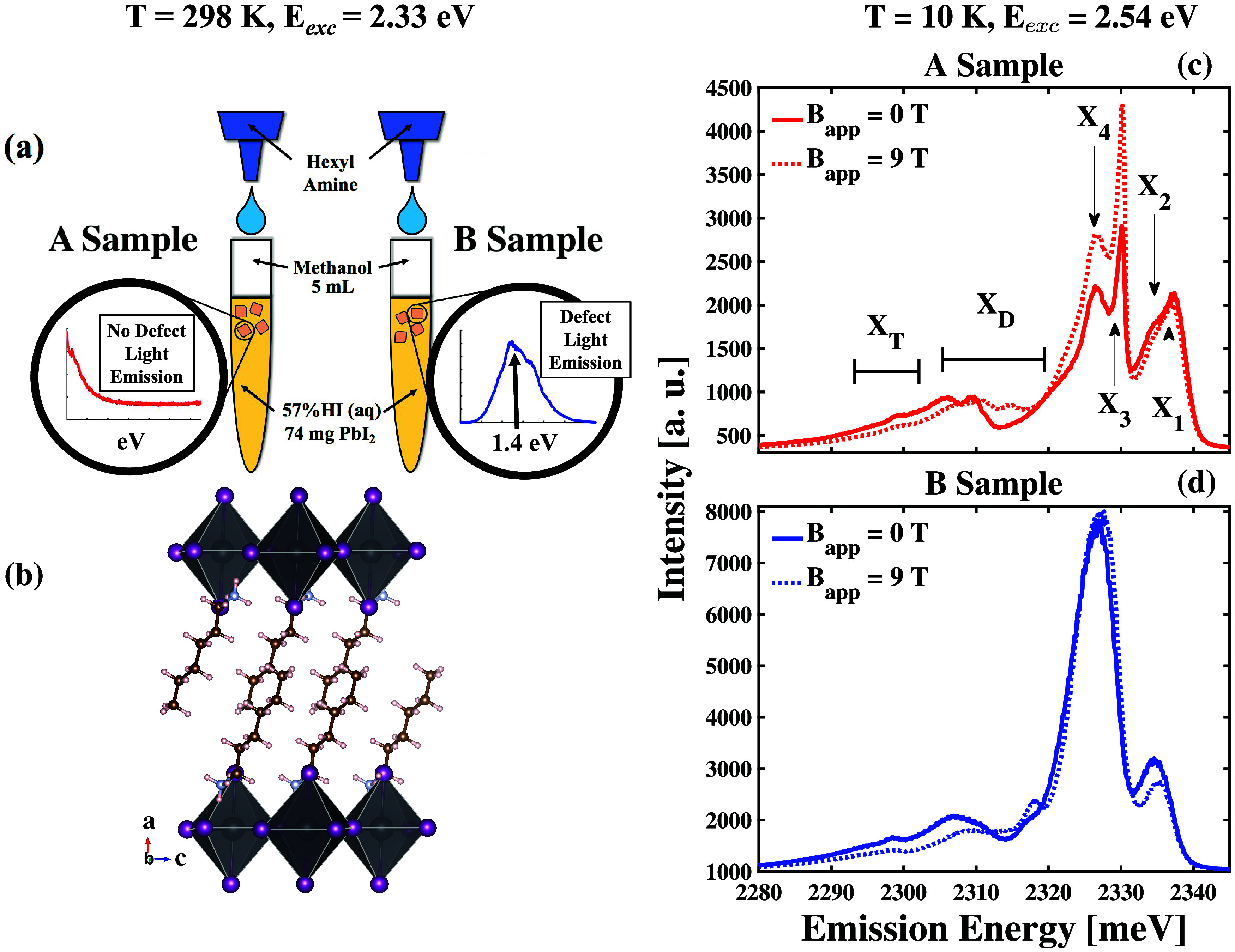
(a) Schematic of the synthetic approaches used in making hybrid organic lead iodide self-assembled quantum wells (SAQWs) to control the presence of broadband photoluminescence at room temperature. Insets: A sample (left) and B sample (right) are photoluminescence (PL) spectra in the subgap spectral region following 2.33 eV excitation at 298 K. (b) Established structure of our hexyl ammonium lead iodide (HA_2_PbI_4_) SAQWs obtained from the synthetic approach described in the text when projected into the material’s *ac*-plane. The crystallographic directions are indicated by colored arrows. (c) Comparison of the PL spectrum of a HA_2_PbI_4_ formed through interfacial solution phase chemistry using nearly stoichiometric ratios of organic and inorganic constituents, denoted as the A sample, measured at 10 K in a parallel-polarized configuration using a 2.54 eV excitation laser under 0 T applied field (solid line) to the PL spectrum measured at the same sample spot under 9 T (dotted line) in a Faraday geometry. Distinct excitonic features (*X*_1_–*X*_4_), defect-associated emission (*X*_*D*_), and trion emission (*X*_*T*_) are labeled. (d) Comparison of the PL spectrum of a HA_2_PbI_4_ formed through interfacial solution phase chemistry using excess organic constituents, denoted as the B sample, measured at 10 K in a parallel-polarized configuration following 2.54 eV excitation under 0 T applied field (solid line) to the PL spectrum measured at the same sample spot under 9 T (dotted line) in a Faraday geometry.

In this structure, the *a*-axis of the crystal unit cell aligns with the direction normal to the inorganic, octahedral layers. These materials possess a bandgap energy near 2.7 eV and excitonic features in absorption spectra near 2.4 eV.^38^ As described in our previous studies, subgap photoluminescence measurements suggest that forming the HA_2_PbI_4_ samples using concentrations of hexylamine in excess of 1:1 stoichiometry drives material self-assembly with incompletely coordinated lead iodide polyhedra.^43^ These chemical conditions lead to the formation of iodide vacancies within the equators of these materials’ octahedral layers, which is consistent with the results of ab initio calculations using supercells containing single vacancy sites.^44^ These differences in defect densities manifest themselves in SEM images of the samples, as shown in Figure S1 and reported in our previous studies.^43^ However, it remains unclear how the application of magnetic fields affects any electronic states caused by defects induced by interfacial solution-solution materials synthesis.

To assess how the magnetic responses of our defective 2D perovskites depend on material synthesis conditions, we formed HA_2_PbI_4_ using two distinct hexylamine concentrations: 0.3 and 1.5 M, which we call the A and B samples, respectively. As detailed in the Methods section below, we determined light emission properties of these samples by placing each sample in a cryostat and cooling them to 10 K prior to taking PL measurements. As shown by comparing the spectra in Figure 1(c,d), we find that forming our samples using the interfacial approach with differently concentrated organic solutions leads to qualitatively different PL spectra following the excitation of free electrons and holes with a 2.54 eV laser.

Figure 1(c) shows the presence of five distinct peaks along with three shoulders on those peaks when we form HA_2_PbI_4_ using organic solutions containing 0.3 M solutions of hexylamine. We note these features in six distinct regions of the spectrum as *X*_1_, *X*_2_, *X*_3_, *X*_4_, *X*_*D*_, and *X*_*T*_ in order of decreasing energy and highlight them in Figure 1(c). Most interestingly, we find that we need to model the *X*_3_ peak at 2330 meV as a Lorentzian function to capture its shape adequately. This Lorentzian shape suggests that the exciton whose recombination gives rise to this peak is unaffected by inhomogeneous broadening under our measurement conditions, despite the fact that we have made no attempts to passivate the sample surface.^56^ Furthermore, we find the *X*_3_ peak possesses a width of 0.64 meV, which is an order of magnitude less than values reported for similar features in the PL spectra of HOIP SAQWs spaced by aromatic ammonium cations.^48,56^ We note that four features have been reported in the PL spectra of phenyl ethylammonium lead iodide at 4 K previously, which we ascribe to the *X*_1_, *X*_2_, *X*_3_, and *X*_4_ peaks in Figure 1(c). Our ability to resolve distinct features in the 10 K PL spectrum of HA_2_PbI_4_ reduces when we consider our more defective B sample. The *X*_1_ and *X*_2_, *X*_3_ and *X*_4_, *X*_*D*_, and *X*_*T*_ features merge into less resolved peaks in the spectrum of the B sample shown in Figure 1(d). We compare the *X*_*D*_ and *X*_*T*_ regions of each sample’s PL spectra measured with a 9 T applied magnetic field in Figure S2. This comparison shows that the B sample emits significantly larger PL intensity throughout the *X*_*D*_ region than does the A sample, which is consistent with the higher defect density we formulated chemically.

The spectra shown in Figure 1(c,d) represent limiting cases in our ability to resolve all of the features in the PL spectra of the HA_2_PbI_4_ A and B samples, respectively. Despite observing a reduced resolution in the different, distinct peaks of the B sample PL spectrum shown in Figure 1(d), we will still find that these general features remain preserved despite the increased defect density relative to the A sample, suggesting the universality of these transitions in the behavior of the material. To assign the underlying electronic excitations corresponding to *X*_*D*_ and *X*_*T*_, we collected PL as a function of the incident laser power, sample temperature, applied magnetic field strength, and the direction in which we applied the magnetic field. PL measurements were also made at different sample locations to assess the variation in the characteristics of spectral peaks and the reproducibility of their magnetic response. We organize the spectroscopic results into different subsections below to assess the specific features present across different regions of each sample and assess their response to the applied magnetic field.

### Power and Temperature-Dependent Photoluminescence Spectra

Figure 2(a) shows the PL spectra of a HA_2_PbI_4_ A sample under the application of a 9 T magnetic field applied in Faraday geometry for different incident laser powers, normalized to the intensity in the region of the *X*_3_ and *X*_4_ peaks. The full data set of power-dependent, normalized PL is shown in Figure S3. Figure S4 shows that the intensities of the *X*_3_ and *X*_4_ peaks vary linearly with incident laser power for all the values that we consider in this study, which suggests that their relative populations do not change as a function of the excitation density we form in the sample. The normalized spectra show that the intensities of features in the *X*_*D*_ region reduce as we increase the laser power, as seen clearly in the upper inset of Figure 2(a). We integrated the PL intensities in the *X*_*D*_ region for all of the powers at which we made measurements and plotted them against laser power, as shown in Figure 2(b). This trend shows a drop in the normalized PL intensity characteristic of defects that become saturated as the excitation density increases.^57^

**Figure 2:**
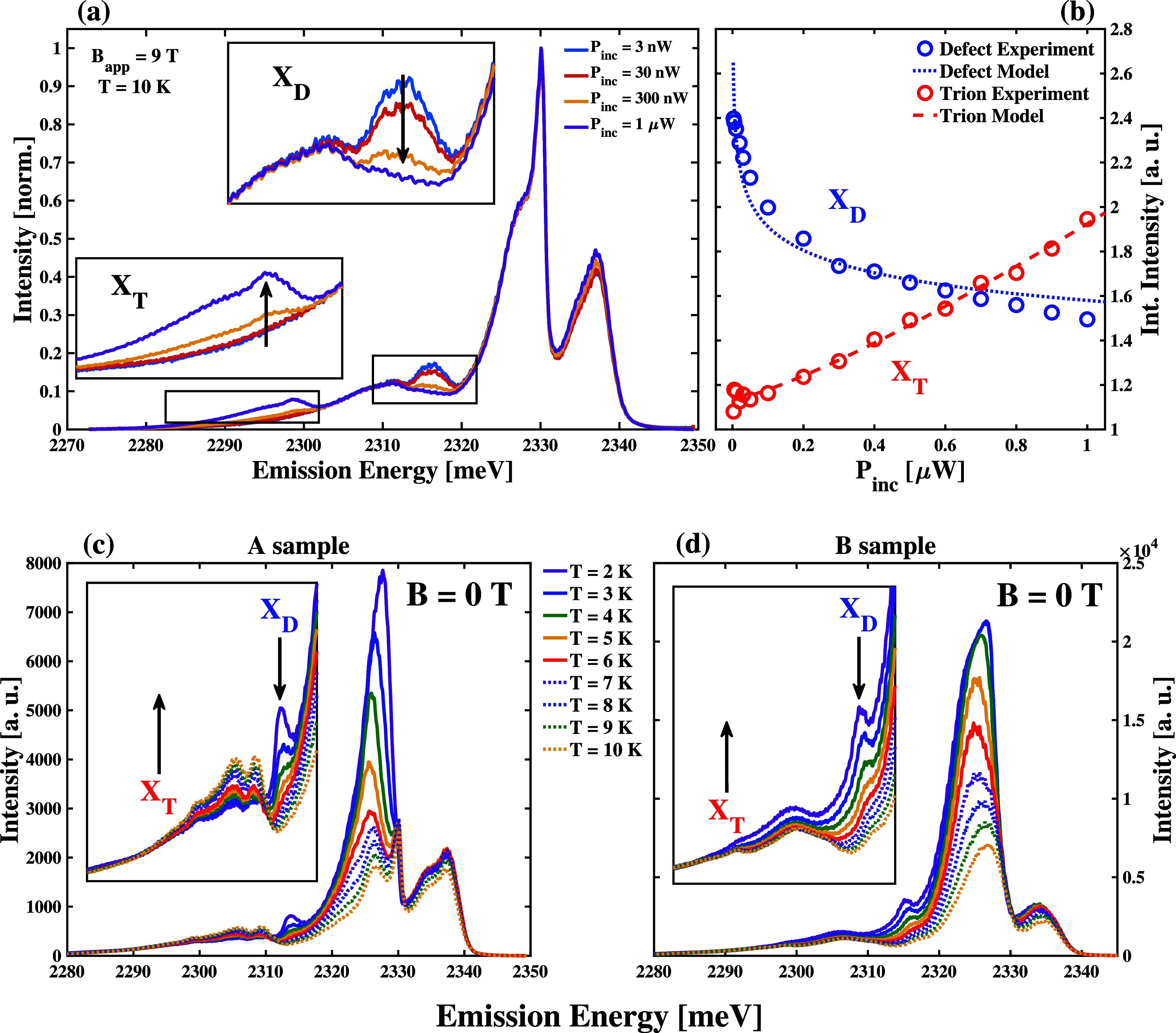
(a) Representative incident, 2.54 eV laser power-dependent PL spectra of a HA_2_PbI_4_ A sample maintained at 10 K while a 9 T magnetic field is applied in the Faraday configuration. These spectra are normalized to the measured PL intensity of the *X*_3_ feature at each incident power. The upper inset zooms in on the X_*D*_ region where the peak intensity saturates with increasing incident laser power, which is consistent with defect filling. The lower inset zooms on the X_*T*_ region where the peak grows with increasing incident laser power, which is consistent with the behavior of trions in semiconductors. (b) Comparison between measured normalized, integrated PL intensities (circles) and the trend developed from kinetic models of semiconductor light emission (dashed line) for the X_*d*_ region (blue) and the X_*T*_ region (red). (c) PL spectra of a HA_2_PbI_4_ A sample measured between 2 and 10 K. The inset zooms in on the region between 2300 and 2320 meV to highlight the temperature dependence of the X_*D*_ and X_*T*_ regions. (d) PL spectra of a HA_2_PbI_4_ B sample measured between 2 and 10 K. The inset zooms in on the region between 2300 and 2320 meV to highlight the temperature dependence of the X_*D*_ and X_*T*_ regions.

Additionally, we find that the normalized intensities of features in the PL spectra below 2300 meV increase as we increase the incident laser power, as shown in the lower inset of Figures 2(a) and S3. Figure 2(b) also shows that the integrated intensity in this low-energy region depends on the incident laser power as *I* ^1.5^, which is consistent with light emission from trion states.^58^ Previous studies show that the trion in phenyl ethylammonium lead iodide (PEA_2_PbI_4_) appears 30–40 meV red-shifted from the main excitonic peak.^59^ These energies are consistent with *GW* calculations for trion states in HOIP SAQWs.^60^ The spectral region that grows as a function of power in the normalized PL spectra lies 35 meV below the *X*_3_ and *X*_4_ peaks, whose contribution to the measurements remains constant, as seen in Figures 2(a) and S3. These facets of the experimental results lead us to propose that the PL signal we measure below 2300 meV results from the recombination of trions, which has not been reported previously in HA_2_PbI_4_.

We only show representative power-dependent spectra in Figure 2(a), which enables us to visualize the trends in the PL signals at the defect exciton and trion energies. Figure S3 shows the spectra that we measure at each power used in our study. These spectra reinforce the trends shown in Figure 2(a). We use the integrated intensities from the spectra in Figure S3 to construct the trends in Figure 2(b) and assign the electronic excitations that give rise to the power-dependent features in our results, as explained above.

Figure 2(c,d) show how the PL spectra of A and B HA_2_PbI_4_ samples depend on temperature between 2 and 10 K when measured at the same positions used to observe the results in Figure 1(c,d), respectively. At 2 K, the *X*_4_ peak is the most intense for both samples. The broad peak that we measure at 2 K narrows significantly as we increase the sample temperatures to 10 K, which allows us to resolve distinct *X*_3_ and *X*_4_ peaks in the A sample PL spectrum.

In addition to changes in the PL spectra near the *X*_3_ and *X*_4_, we find a prominent, but less intense peak in the X_*D*_ regions of the 2 K spectra of both samples. As we heat the samples to 10 K, the intensity of this peak decreases significantly to the point that we do not observe it distinctly. We highlight this temperature dependence with arrows in the insets of Figure 2(c,d). These arrows show the decreased intensity of the X_*D*_ region in the PL spectra of both our A and B samples as we increase the sample temperature from 2 to 10 K using downward arrows. Additionally, we highlight the increased prominence of the X_*T*_ region as we increase the samples’ temperatures from 2 to 10 K using upward arrows.

Previously, we observed similar behavior to that seen in Figure 2(c,d), corresponding to competition between narrow and broadband light emission features near 2000 meV.^44^ The temperature dependence of that competition was consistent with reorganization of the defect state along a vibrational mode of the hexyl ammonium molecular cation.^44^ Based on these previous results, we propose that the temperature dependence of the 2314 meV peak intensity suggests that thermal activation over a small energy barrier is necessary to depopulate the emissive state at temperatures of 10 K and above. We consider the magnetic response of these features in the Results and Discussion section below when assessing this hypothetical proposal.

The power and temperature-dependent PL spectra of our HA_2_PbI_4_ samples indicate that defect states lead to light emission features between 2310 and 2320 meV, which have not been reported previously. Additionally, the power dependence and energies of PL intensity between 2290 and 2300 meV are consistent with those of trions in other HOIP materials. We assessed the response of these features to varying applied magnetic fields to better understand the fundamental properties of underlying states and work toward their control for optoelectronic applications. Since the powers used in our PL measurements on the A sample significantly reduce the intensity of PL from the *X*_*D*_ region for conditions where we observe the trion features, we will focus on the magnetic response of the HA_2_PbI_4_ B samples to determine the magnetic properties of both types of excitation simultaneously. These samples have higher defect densities such that we cannot saturate the PL intensity of the *X*_*D*_ at incident laser powers where we observe an appreciable signal in the *X*_*T*_ region.

### Magneto-Photoluminescence Spectra of HA_2_PbI_4_ B Sample

The panels of Figure S5 compare the PL spectra in the *X*_*T*_ and *X*_*D*_ regions of our HA_2_PbI_4_ B sample measured at four distinct positions, Spot 1, Spot 2, Spot 3, and Spot 4, under the application of no magnetic field to those observed with a + 9 T field applied in a Faraday configuration. These comparisons show that these different spots of our sample behave in qualitatively similar ways.

With an applied field, we observe a reduction in the PL intensity below 2310 meV and an increase in PL intensity above 2315 meV in all of these spectra. Based on the assignments given in Figure 1(c), these field-induced changes in the PL spectra indicate that the intensity emitted by the defect states in the *X*_*D*_ region increases while the signal we observe by trion states in the *X*_*T*_ decreases as we increase the applied field strength. Interestingly, we find that the prominence of a distinct, narrow peak around 2298 meV reduces significantly in the spectra we measure at Spot 1, Spot 2, and Spot 4 as we increase the magnetic field applied to the sample. These similarities indicate that the features of the PL spectra of this sample are affected by application of a magnetic field in the same way, even in the presence of some variation in the specific structure of each spot’s respective spectrum.

Figure S6 compares the PL spectra of our HA_2_PbI_4_ B sample measured at Spot 1 at 10 K under a 0 T applied magnetic field to the contributions we model with energies below 2300 meV. Given the ∼1.5 exponentially increasing intensity we measure at these energies in the PL spectra of our A sample upon raising the incident laser power, shown in Figure 2(b), we propose that these features correspond to trions in the samples, which we explained above. As seen in Figure S6, the two peaks’ widths differ significantly, which we attribute to differences in their nature.

We observe a narrower peak at the same energy (2298 meV) in three of the spectra shown in the panels of Figure S5 (Spots 1,2, and 3), which we denote as the *X*_*T*_1__ state. The feature we assign to this state is shown as a red Gaussian peak defined by the parameters we extract from the models used to reproduce the experimental spectrum. We denote the broader peak as *X*_*T*_2__ and note that this feature shifts significantly as a function of the spot where we illuminate the sample. We find this peak near 2295 meV in the spectra measured at Spot 1 and Spot 4, but we find our models need to shift this peak to the vicinity around 2305 meV to explain the spectra we measure at Spots 2 and 3. The difference in the variation of each peak’s position indicates that the energies of the *X*_*T*_1__ and *X*_*T*_2__ states differ in their sensitivities to external perturbations, as discussed in more detail below.

As seen by our experimental results, the structures and intensities of the HA_2_PbI_4_ PL spectra that we measure in the *X*_*D*_ and *X*_*T*_ regions respond systematically to the application of a magnetic field. However, the mechanisms driving these changes are not evident from the raw data. We used our fitted spectra to assess these physical mechanisms in the *X*_*D*_ and *X*_*T*_ regions separately.

#### Assessing Zeeman Coupling to *X*_*D*_ States

As seen in Figure S5, a peak appears at 2317 meV in the PL spectra corresponding to each spot of the B sample when applying a 9 T field. We fit the positions of this peak found at the B sample Spots 1 and 2 as a function of applied field strength to model functions of spectra measured at both sample spots. These trends are shown in Figure 3. We then fit those field-dependent trends to linear models to approximate Zeeman coupling as a function of the effective magnetic field strength, which we denote as μ_B_B_app_ in units of meV, i.e., *E*_*D*_*i*__ = [*g*_*e*,_ *_i_* – *g*_*h*,*i*_]­μ_B_B_app_ = *g*_ex_μ_B_B_app_. As shown by the comparison between experimental and model results, we find nearly identical linear shifts in the *X*_*D*_ peak positions measured at both spots and estimate *g*_1_ = 1.8 ± 0.1 and *g*_2_ = 2.1 ± 0.3. Similar *g*_ex_-factor values were reported previously for the magnetic response of in-plane, free excitons of phenyl ethylammonium lead iodide (PEA_2_PbI_4_) at 4 K.^48^ The fact that we find equal *g*-factor estimates within the uncertainty of our fitting approach supports the conclusion that we observe the same defect states in the spectra measured at both sample spots. Figure S7 shows a comparison of *E*_*D*_ across the four spots that we assessed in this study.

**Figure 3:**
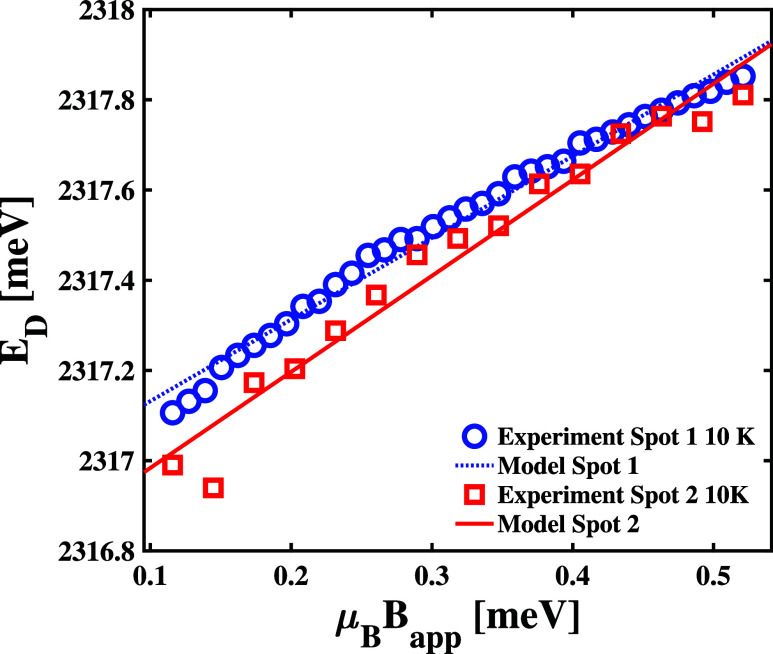
Comparison between the measured magnetic field-dependent positions of the *X*_*D*_ peaks at Spot 1 (°) and Spot 2 (□) to Zeeman model fittings, which correspond to the solid and dashed lines, respectively.

The panels in Figure S8 show measurable changes in the PL magneto-PL spectra of our B sample at Spot 3 when we cool the material from 10 to 1.6 K. To test this hypothesis, we plotted a comparison of the position of the 2314 meV peak as a function of the applied magnetic field in Figure 4. This comparison shows that the peak position shifts linearly with field strength for the spectra measured at 1.6 K, but does not shift within the uncertainty of our model fits for measurements made at 10 K. We use the linear fit to estimate that *g*_ex_ = 0.16 ± 0.01 at 1.6 K and *g*_ex_ = −0.01 ± 0.03 at 10 K. The change in the magnetic response of this peak as we modulate the sample temperature may suggest an increased localization of effective charge at the defect site at lower temperatures. As seen in Figure 2(d), the intensity of the peak at 2314 meV increases significantly as we cool the sample to 2 K, even when measuring the PL spectrum at Spot 1. If this intensity increase results from a larger steady-state population within the defect site at 2 K, then that increased population could bring with it a larger effective charge, which accounts for the linear magnetic response we show in Figure 4. This physical picture would be consistent with a reduced structural reorganization of the crystal lattice at lower temperatures, which we observed previously in the case of midgap defect states formed in HA_2_PbI_4_ samples grown at liquid–liquid interfaces.^44^

**Figure 4:**
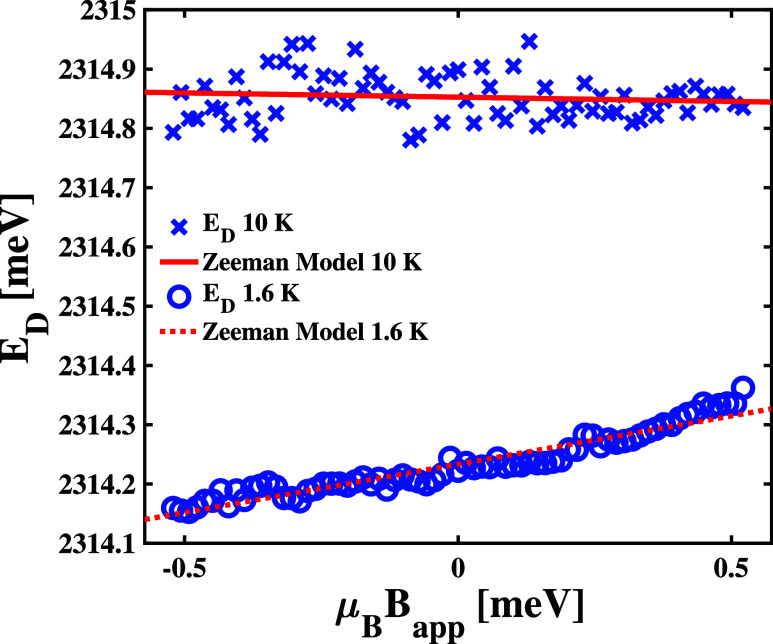
Comparison between the measured magnetic field-dependent positions of the *X*_*D*_ peak at Spot 3 of the HA_2_PbI_4_ B sample at 10 K (×) and 1.6 K (°) to Zeeman model fittings, which correspond to the solid and dashed lines, respectively.

We used DFT calculations to complement our understanding of the magnetic properties of defect states in our HA_2_PbI_4_ samples. In one case, we calculated the electronic band structure of pristine HA_2_PbI_4_, as detailed in the Methods section. We show the atom-resolved PDOS extracted from these calculations in Figure 5(a). This visualization shows that the valence bands of the pristine material form from the p_*x*,*y*,*z*_-orbitals of I^–^ and s-orbitals Pb^2+^, while the conduction band is composed of the p_*x*_- and p_*y*_-orbitals of Pb^2+^, respectively. Previous studies have established that these are the compositions of the band edges pertinent to photoexcitation.^61^

**Figure 5:**
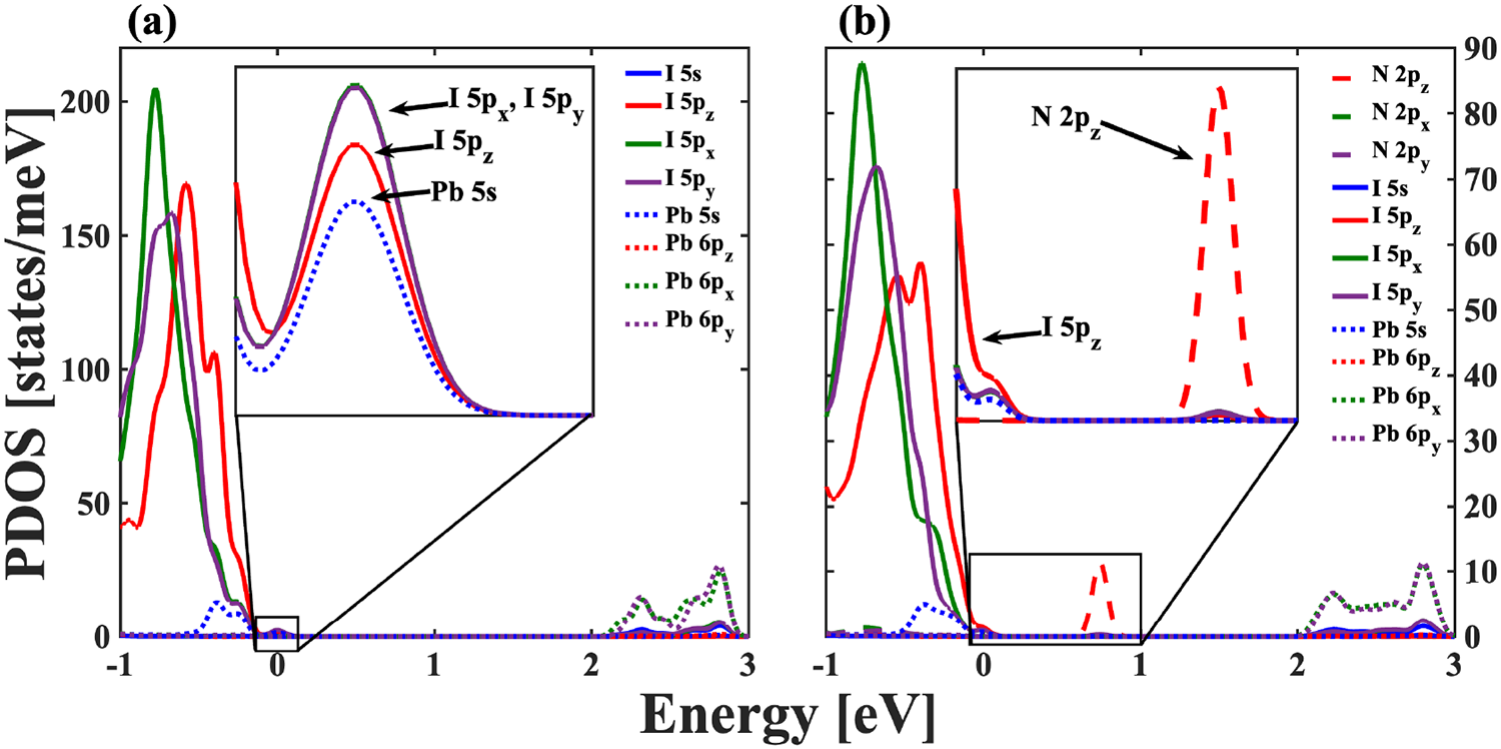
Comparison between the atom-resolved PDOS of pristine (a) and defective (b) supercells of HA_2_PbI_4_ found from DFT calculations. Insets in each panel show expanded views of the atomic compositions of the valence band edge and low-energy midgap region of the PDOS of each material type, which indicates the reduced angular momentum of holes in the defective electronic structure relative to that of the pristine system.

In addition to these calculations of the pristine band structure, we also assessed the electronic states in the presence of a correlated vacancy of an equatorial I^–^ site and H^+^ off the molecular cation. Based on our removing these ions from a 2 × 2 × 1 supercell of the material, we produce a nearly 6% doping of the crystal, which is likely much more than actually produced in our experiments. However, we find that this level of doping produces flat electronic bands within the material’s bandgap, which would indicate spatially localized electronic states consistent with those introduced by dilute defect sites. Our previous studies of narrowband light emission in these systems indicate that these defects introduce electronic states inside the material’s bandgap that are consistent with subgap PL spectroscopic results.^44^ Based on this observation, we propose that the defect doping is small enough to help explain the salient features of our experimental measurements.

As shown in Figure 5(b), the introduction of this correlated defect site results in the appearance of an electronic state ∼0.25 eV above the valence band edge. The PDOS associated with this state indicates that it results predominantly from the p_*z*_ orbital of the N atom on the molecular spacer and some contribution from the I^–^ p_*y*_ orbital. The nitrogen orbital possesses a zero projection of the orbital angular momentum onto the *z*-direction, which would correspond to the *a*-axis of our HA_2_PbI_4_ sample. Additionally, the extraction of the I^–^ from the equator of the inorganic plane changes the composition of the valence band edge. The inset in Figure 5(b) shows that the contributions from the p_*x*_ and p_*y*_ orbitals of the I^–^ decrease at the VB edge relative to the case of the pristine material. This reduction in their contribution to the band edge coincides with an increased amount of the p_*y*_ orbitals of the I^–^ surrounding the defect site, contributing to its electronic structure, as seen in the inset of Figure 5(b). This fact would suggest that the magnetic response of an exciton trapped near that site would not differ significantly from that of the free excitons, which carry the nonzero orbital angular momentum from these I^–^ states through their contribution to the valence band in the pristine regions of the material, as reported previously.^61^ Based on this physical picture, we expect that the atomic composition of the defect should maintain *g*_ex_-factor values similar to those found in pristine regions of the material. These assessments lend support to our findings that the energies of *X*_*D*_ measured at Spot 1 and Spot 2 of the HA_2_PbI_4_ shift in response to the applied magnetic field with values of *g*_ex_-factor similar to those reported previously for free excitons in PEA_2_PbI_4_ at 4 K.^48^ As shown in Figure S9, incorporating the effect of spin–orbit coupling into our calculations of the defective band structure does not change the properties of the localized defect state significantly.

**Figure 6:**
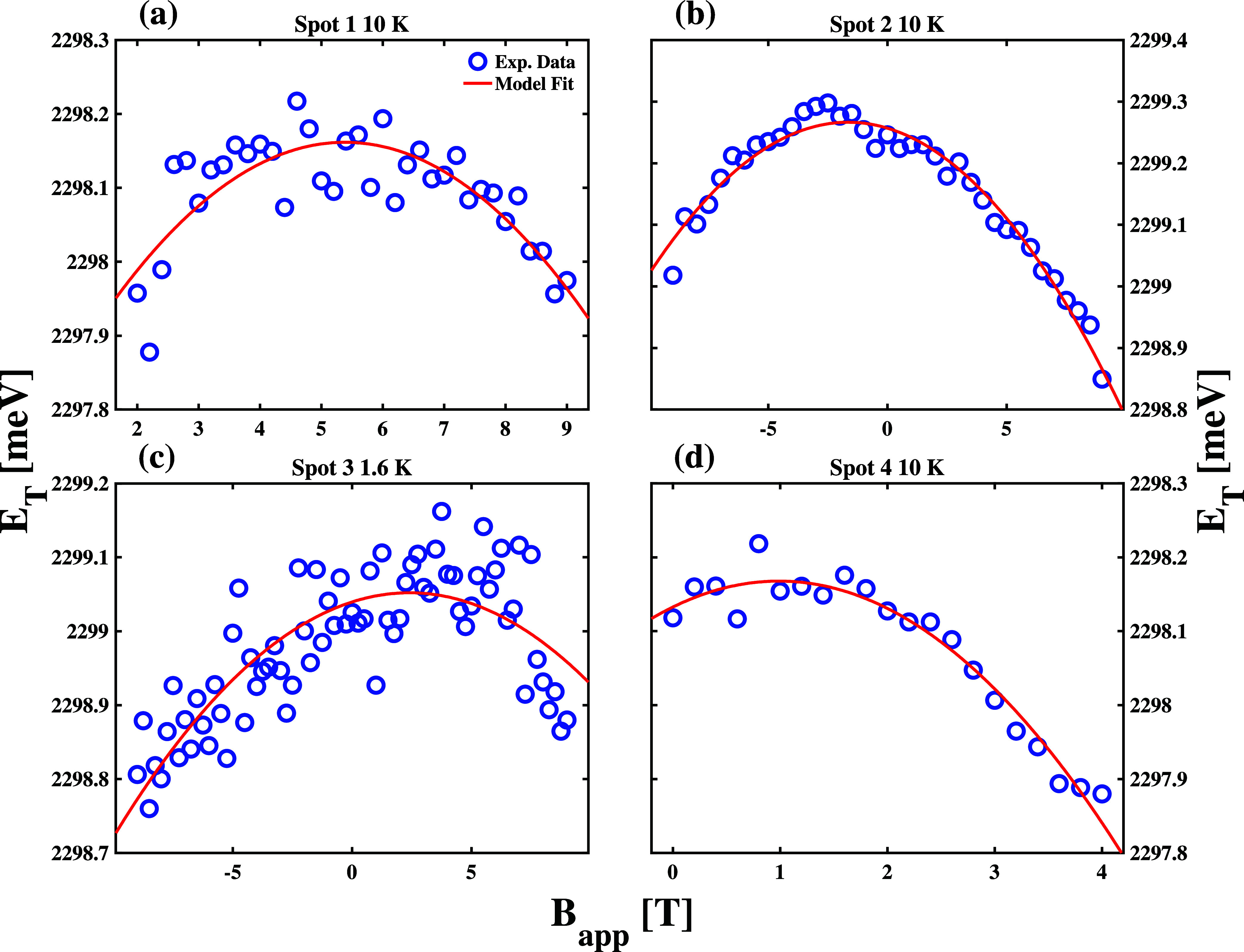
Comparison of the magnetic responses of the PL signals from the *X*_*T*_1__ states measured at Spot 1 (a), Spot 2 (b), Spot 3 (c), and Spot 4 (d) of the HA_2_PbI_4_ B sample. Note that the sample was held at 1.6 K for the spectrum measured taken at Spot 3 while all other spectra were acquired at sample temperatures of 10 K.

### Magnetic Response of Trion States

We noted above that we find two features in the models of our HA_2_PbI_4_ PL spectra whose power dependence matches that we expect for trion states: a narrow feature we assigned to *X*_*T*_1__ and a broader contribution that we denoted *X*_*T*_2__. In addition to their different widths and energies as a function of the spot where we collect the PL measurement, we find differences in their responses to the applied magnetic field.

The panels of Figure 6 compare the energies of *X*_*T*_1__ (*E*_*T*_1__) that we measure at the four spots of the *B* sample as a function of applied magnetic field to model fits of the experimental results. For each of the spots, we find that the *E*_*T*_1__ values change quadratically with the strength of the applied field. Additionally, we find that the highest value of *E*_*T*_1__ does not appear at the same magnetic field amplitude for each sample spot. However, we find that the quadratic parameter of our model fits does not vary significantly as a function of the sample spot, which we show in Table 1.

**Table 1:** Parameter Estimates from Fits of the Experimental Shifts of *E*_*T*_1__ to the Equation *E*_*T*_1__ = *A*(*B* – *B*_offset_) ^2^ + *E*_0_ as a Function of Spot Illuminated in PL Measurements

spot	A [meV/T^2^]	*B*_offset_ [T]	*E*_0_ [meV]
1	–0.015 ± 0.004	5.4 ± 0.3	2298.1 ± 0.1
2	–0.0035 ± 0.0003	–1.6 ± 0.3	2299.3 ± 0.1
3	–0.0021 ± 0.0006	2.4 ± 0.9	2299.1 ± 0.1
4	–0.036 ± 0.008	0.99 ± 27	2298.2 ± 0.1

The inverted parabolic behavior of *E*_*T*_1__ does not conform to standard theories of the magnetic response of isolated electronic excitations. However, we find that this surprising behavior is observed robustly at each sample spot where we make our PL measurements, which indicates that it represents a universal and fundamental property of the sample. We expect that any quadratic response in our material to correlate positively with the applied magnetic field, as seen in the standard diamagnetic response of excitons reported previously for some HOIP SAQWs.^45,50^ In contrast, recent studies indicate that interactions between excitons and excitonic complexes in 2D materials can lead to anomalous, quadratic shifts in peak energies that correlate negatively with the applied field strength.^52,54,62^

For example, Ma et al. used restricted band, tight-binding calculations to consider how exchange interactions between the singlet and triplet levels of trions in the TMDC WeS_2_ determine how this 2D material responds to applied magnetic fields.^52^ These authors find that the applied magnetic field changes the detuning between the singlet and triplet states, which determines their energies as a function of the applied field and the identity of the emissive bright state in PL measurements. By considering different electron exchange coupling energies, Ma et al. also find that PL observables depend on the applied magnetic field nonlinearly, with maxima that do not align with the zero-field case but rather indicate the magnetic field strengths that drive the singlet and triplet states into resonance. The nonlinear magnetic response then corresponds to an avoided crossing behavior between the two states that are coupled by exchange interactions. Since one of the two states remains dark, only the behavior of one state manifests itself in the experimental results. Similar results have been observed in the magnetic response of excitonic light emission in the layered magnetic system CrSBr and were attributed to magnetically controllable interactions between spin states of excitons and surrounding magnetic excitations.^54^

Based on these previous studies, we propose that the observed negative quadratic response of *E*_*T*_1__ results from interactions between the trion states and other electronic excitations whose energies lie close to that of *X*_*T*_1__. As noted above and shown in the panels of Figure S6, the *X*_*T*_1__ and *X*_*T*_2__ features overlap energetically in the PL spectra we observe at Spot 1 of our B sample. The spectral proximity of these two features would induce faster transitions between the two states in the presence of any interactions. However, when we consider the magnetic response of *E*_*T*_2__ shown in Figure S10 for the measurement at Spot 1, we do not observe any indication of an avoided crossing. Rather we find a linear modulation of the peak energy consistent with a Zeeman effect between *X*_*T*_2__ and the applied magnetic field. This finding suggests that interactions between *X*_*T*_1__ and a dark excitonic state lead to the behavior we observe in the panels of Figure 6.

The magnetic field offset, *B*_0_, needed to induce the resonance between *X*_*T*_1__ and another dark state would also depend sensitively on the exact detuning between the interacting states at each specific sample spot. As seen in Table 1, we find a negative *B*_0_ only for the PL measurement at Spot 2, where *E*_0_ has the largest value. This fact suggests that a positively sloped magnetic shift is needed to drive *X*_*T*_1__ into resonance with another state that remains dark in our measurements. In the presence of smaller zero-field interactions, the magnetic field strength necessary to induce resonance between the interacting groups would shift to negative values while maintaining the same overall shape as a function of *B*_app_. Restricted band, tight-binding calculations using Coulombic interactions between electrons and holes will be needed to determine what interaction with specific states could explain our experimental results, but are beyond the scope of this study.

## Conclusions

In conclusion, we have used low-temperature photoluminescence spectroscopy to assess how excitons and excitonic complexes respond to applied magnetic fields in defective 2D hybrid organic–inorganic self-assembled quantum well samples grown at liquid–liquid interfaces. Unlike samples of these materials grown via bulk solution methods, we find that we can resolve Lorentzian peaks in the light emission of interfacially formed species without mechanical exfoliation or encapsulation with additional low-dimensional materials. We use variations in the applied laser power, changes in the sample temperature, and applied magnetic fields to assign the peaks in the low-energy regions to excitons trapped at defects and trions in these systems. We find that the defect excitons respond to applied magnetic fields in the same manner as charged excitations and estimate *g*_ex_-factor values that match those reported for “free” excitons in previous studies.^48,56^ We leverage ab initio calculations of the electronic structures of pristine and defective structures of our samples to motivate a rationale for the observation of these magnetic interactions in our defective HOIP SAQW samples. Additionally, we observe anomalous magnetic response from peaks we assign as trions in the PL we measure at multiple spots on our defective HOIP SAQW sample. We propose that this response stems from interactions between the trion and other excitons or excitonic complexes that remain dark in our experiments. These results indicate that liquid–liquid interfacial synthetic methods could play an important role in forming specific defective structures in HOIP SAQW samples that enable control over the magnetic response of these materials’ excitonic excitations, which could be useful for the application of these materials in photon-based QIS technologies.

## Methods

### Materials Synthesis

We purchased all chemicals from Sigma-Aldrich and used them as received without further purification. To synthesize the SAQW samples, we modified a layered approach reported previously.^63–65^ This method involves layering two solvents with different densities to create an interface where precursors slowly interact by diffusion and form the SAQW materials.

We used this layered synthetic method to form both pristine and highly defective crystals of hexyl ammonium lead iodide (HA_2_PbI_4_). Specifically, to produce more pristine crystals of HA_2_PbI_4_, we dissolved 74 mg of PbI_2_ in 1.5 mL of 57% hydriodic acid (HI) in a beaker and transferred gently into a test tube (18 mm × 150 mm) using a syringe. To the test tube containing PbI_2_ in HI solution, we gently added 5 mL of methanol. Due to the difference in densities, an interface was formed between methanol and HI. We added 0.2 mL of hexylamine to the test tube using a syringe, which leads to an amine concentration of 0.3 M within the methanol solvent. We covered the test tube with aluminum foil. Orange plate-like crystals formed between 2 and 3 weeks after adding the materials to the test tube. We filtered the orange crystals by suction, washed them with cold diethyl ether, and dried them in an oven at 60 °C for 24 h. We refer to those materials made through this set of chemical conditions as more pristine or A samples.

To synthesize more defective HA_2_PbI_4_ SAQW samples, we followed a method similar to that used in making the A samples but with a slight modification. While we prepared the layered aqueous/methanol solutions in the same fashion as the case of our A samples, to form more defective SAQWs we added 1 mL of hexylamine to the test tube, which leads to a concentration of 1.5 M in the methanol solvent. After covering the test tube with aluminum foil, we obtained orange plate-like crystals within 3 days, which we isolated using suction filtration after 2 weeks. We washed the orange crystals with cold diethyl ether and dried them in an oven at 60 °C for 24 h. As proposed in our previous studies, the chemical equilibrium controlling SAQW formation can be driven toward the product side by increasing the hexylamine concentration.^43^ The increased rate of product formation results in materials synthesis using incompletely coordinated Pb–I polyhedra, which template I^–^ vacancies in the equatorial position of the inorganic layers of the SAQWs. We corroborated this picture of materials chemistry using computational methods previously.^44^ We refer to the materials made under these chemical conditions as our B samples.

### Single-Crystal X-ray Diffraction Structural Characterization

A suitable orange crystal (0.01 × 0.05 × 0.10) mm^3^ was mounted on a MicroMount (MiTeGen) with paratone oil (Parabar 10312, Hampton Research) on a Bruker D8 Venture diffractometer with kappa geometry, an Incoatec IμS microfocus source X-ray tube (Mo Kα radiation), and a multilayer mirror for monochromatization. The X-ray diffraction intensities were measured by using a Photon III CPAD area detector at a distance of 50 mm. Data were acquired at 100 K with an Oxford 800 Cryostream low-temperature apparatus. The intensities were integrated using SAINT V8.41, and a multiscan absorption correction was applied with TWINABS with APEX6 v2024.9–0. The crystal structure was solved using a dual-space approach as implemented in SHELXT^66^ and difference Fourier (Δ*F*) maps during least-squares refinement, as embedded in SHELXL-2019/3 ^67^ running under Olex2.^68^ All non-hydrogen atoms were refined anisotropically. Hydrogen atoms were positioned with idealized geometry and refined isotropically using a riding model. At 100 K, the structure was refined in the centrosymmetric group *P*2_1_/*c* with 0.5 molecules in the asymmetric unit and *Z* = 2. The crystal was determined to be a two-component nonmerohedral twin related to each other by a 180° rotation around the direct [1 0 0] rotation vector. Final refinement was performed using the HKLF-5 with reflections from both domains, which led to a batch scale factor (BASF) parameter of 0.3081(12). Crystal data were deposited in the Cambridge Crystallographic Data Centre with number 2498631.

### Photoluminescence Measurements

Photoluminescence (PL) spectra were obtained with a 488 nm (2.54 eV) excitation from an argon ion laser in the 180° backscattering configuration using a dispersive spectrometer (Horiba JY T64000, 1800 groove/mm grating), which is coupled to a liquid-nitrogen-cooled CCD detector. To perform low-temperature, magnetic field-dependent PL spectroscopy, the HA_2_PbI_4_ A and B samples were adhered to a Si(100) substrate and placed into an attoDRY 2100 cryostat (attocube Inc.). The sample holder was pumped to ∼7 × 10^–6^ Torr, backfilled with helium gas, and cooled. We illuminated micrometer-sized flakes with a low-temperature, magnetic field-compatible microscope objective (50×, N.A. = 0.82), which produced laser spot sizes near 1 μm. After moving the samples to appropriate positions using *xyz* nanostages, we used integration times of 10 s and the laser powers between 3 nW and 3 μW to acquire PL spectra at constant applied magnetic fields. Spectral acquisition at these powers reduced local sample heating. Spectra acquired with applied magnetic fields were corrected for Faraday rotation in the objective using automated and motorized achromatic half-wave plates external to the magneto cryogenic system. For relative polarization-dependent PL measurements, we controlled both the incident and emitted light field polarization states using ultra-broadband polarizers and achromatic half-wave plates. We used a neutral density filter to adjust the flux of the incident laser source to make power-dependent measurements on our HA_2_PbI_4_ A sample while maintaining a 9 T applied magnetic field.

### Electronic Structure Calculations

We use first-principles density functional theory (DFT)^69,70^ to assess the electronic structure of pristine and defective HA_2_PbI_4_, as detailed previously.^44^ From these calculations, we evaluated the projected density of states (PDOS) for two separate systems: (a) the pristine structure and (b) a defective structure. Previous results show that DFT calculations of the PDOS with additional defects do not produce states consistent with light emission spectra that are observed experimentally.^44^ We employ the Quantum ESPRESSO package^71^ for all the calculations reported in this study using the optimized norm-conserving Vanderbilt (ONCV) pseudopotentials.^72^ Spin–orbit coupling (SOC) effects were included through the noncollinear formalism as implemented in the Quantum ESPRESSO package^71^ and fully relativistic pseudopotentials.^72,73^ Previously we found that the 2 × 2 × 1 supercell yields almost the same results as the 3 × 3 × 1 supercell.^44,74^ Based on this previous finding, we use the 2 × 2 × 1 supercell size to understand the magnetic response of HA_2_PbI_4_ in this work.

## Supplementary Material



## Notes

Details related to sample scanning electron micrographs, the analysis methods used to fit PL spectra, power-dependent PL spectral model, spatial variation in PL spectra and magnetic responses, and band structures of defective models computed with DFT (PDF)
