# Supplementary material for: Magnetic Response of Excitons and Excitonic Complexes in Defective Hexyl Ammonium Lead Iodide Self-Assembled Quantum Wells
Source: ACS Nano. 2026 Mar 27;20(14):10794–806. doi: 10.1021/acsnano.5c07593 (PMC13085914; doi:10.1021/acsnano.5c07593)
Supplement: Supplementary file 1 [file nn5c07593_si_001.pdf]

Supporting Information for Magnetic Response of Excitons and Excitonic Complexes in Defective Hexyl Ammonium Lead Iodide Self-Assembled Quantum Wells

Maria F. Munoz,† Destiny Konadu,‡ Adedayo M. Sanni,‡ Casandra L. Ward,¶ Atish Ghosh,‡ Amos Afugu,‡ Zhen-Fei Liu,‡ Angela R. Hight Walker*,† and Aaron S. Rury*,§,‡

†Quantum Measurement Division, National Institutes of Standards and Technology, Gaithersburg, MD, US 20899

‡Department of Chemistry, Wayne State University, Detroit, MI, USA 48202

¶Lumigen Instrument Center, Wayne State University, Detroit, MI, USA 48202

§Materials Structural Dynamics Laboratory, Wayne State University, Detroit, MI 48202

E-mail: angela.hightwalker@nist.gov;arury@wayne.edu

Contents

List of Figures **S2**

S1 Structural Characterization of Hexyl Ammonium Lead Iodide using Single Crystal X-ray Diffraction **S4**

S2 Scanning Electron Micrographs of Hexyl Ammonium Lead Iodide Samples **S5**

S3 Analysis of Photoluminescence Spectra **S6**

S3.1 Comparison of Magneto-Photoluminescence Spectra of A and B Samples S6

S3.2 Power Dependent-Photoluminescence Spectra S7

S3.3 Spatial Inhomogeneity of X_D and X_T PL Features in HA_2PbI_4 B Sample S9

S3.4 X_{T_1} and X_{T_2} peaks in B Sample PL Spectra. S9

S3.5 Variation of X_D g-factors S10

S3.6 Temperature dependence of X_D features at Spot 3 of HA_2PbI_4 B sample S10

S3.7 Incorporating Spin-Orbit Coupling in Electronic Structure of Localized Defect States S12

S3.8 Spatial Variation in E_{T_2} peak energies in B Sample PL Spectra. S14

References **S16**

List of Figures

S1 Comparisons of the SEM images of hexyl ammonium lead iodide A (panels a and c) and B (panels b and d) samples showing differences in their morphologies due to distinct defect concentrations. S5

S2 Comparison between the X_D and X_T regions of the A sample (blue) and B sample (red) PL spectra measured with a 9 T magnetic field applied in the Faraday configuration. S7

S3 Incident laser power dependence of the normalized PL spectra we measure at spot 1 of the HA_2PbI_4 A sample. The insets highlight the behavior of the PL intensity in the regions of the X_D and X_T features. S8

S4 Incident laser power dependence of spectral features extracted from models of the HA_2PbI_4 A sample PL spectra corresponding to the X_4 exciton (a) and X_3 exciton (b) when measured at 10 K . Insets: comparison between the power dependence of the integrated intensity of the X_4 (a) and X_3 (b) features measured experimentally (blue squares) at 10 K to linear fits (dashed red) on the log-log scale. S9

S5 Comparison between the PL spectra of a HA_2PbI_4 B sample measured at 10 K with 0 T applied in a Faraday configuration (red) to that measured with 9 T applied in a Faraday configuration at Spot 3 while holding the sample temperature at 10 K (a) and 1.6 K (b). Magneto-PL difference spectra constructed by subtracting the spectrum measured at Spot 3 for 0 T applied magnetic field from that measured at 9 T while holding the HA_2PbI_4 B sample at 10 K (c) and 1.6 K (d). S10
S6 Comparison between the PL spectrum of our HA_2PbI_4 B sample measured 10 K under 0 T applied magnetic field (solid black) to X_{T_1} (filled red) and X_{T_2} (filled blue) contributions to models used to explain the experimental results. Features at higher energy than the X_{T_1} and X_{T_2}states lie in the X_D S11
S7 Comparisons of the experimental PL emission peak energy of the X_D exciton of the HA_2PbI_4 B sample as a function of applied magnetic field strength measured at 10 K (blue circles) to fits of these data to Eqn. (S2) (red line). S11
S8 Comparison between the PL spectra of a HA_2PbI_4 B sample measured at Spot 3 with -9 T applied in a Faraday configuration (red) to that measured with +9 T applied at 10 K (a) and 1.6 K (b). S12
S9 Electronic band structure of a defective HA_2PbI_4 supercell including the effects of spin-orbit coupling. The reader should note minimal changes in the characteristics of the defect state, which results from contributions by N p_z and I p_y atomic orbitals.S13
S10 Comparisons of the experimental PL emission peak energy of the X_{T_2} exciton of the HA_2PbI_4 B sample at spot 1 (top) and spot 4 (bottom) as a function of applied magnetic field strength at 10 K (circles) to fits of these data to linear (top, solid line) and nonlinear models (bottom, solid line). S15

S1 Structural Characterization of Hexyl Ammonium Lead Iodide using Single Crystal X-ray Diffraction

Table S1 reports the structural characteristics of our HA_2PbI_4 A sample established by single crystal X-ray diffraction measurements at 100 K.

Lattice parameters of HA_2PbI_4 extracted from single crystal X-ray diffraction measurements at 100 K: Space group: monoclinic *P2$_1$/a*; *a* = $16.0937(13)$ Å, *b* = $8.8240(6)$ Å, *c* = $8.6225(5)$ Å, *V* = $1223.61(15)$ Å^3; $\alpha = \gamma = 90°$; β = 92.169(16)°; non-hydrogen atom positions are reported in fractional coordinates and hydrogen are reported in Å^4.

Compound	Atom	*x*	*y*	*z*	U_{iso}
$(C_{12}H_{32}N)_2PbI_4$	Pb1	0	5000	0	12.19(12)
	I1	30.3(5)	6938.3(7)	3081.1(7)	16.79(15)
	I2	1990.8(4)	4689.6(7)	287.3(8)	17.66(15)
	N1	1597(6)	4279(11)	4317(10)	21(2)
	C1	1989(7)	5415(11)	5390(13)	20(2)
	C2	2903(8)	5081(13)	5646(14)	24(3)
	C3	3323(7)	6248(14)	6682(13)	24(2)
	C4	4210(8)	5855(13)	7140(15)	26(3)
	C5	4644(8)	6966(14)	8247(15)	29(3)
	C6	5535(8)	6588(16)	8676(17)	34(3)
	H1A	1857.95	4293.29	3399.04	25
	H1B	1050.28	4509.39	4149.45	25
	H1C	1644.57	3340.09	4747.88	25
	H1D	1712.77	5389.42	6395.7	23
	H1E	1917.04	6442.8	4944	23
	H2A	2972.03	4065.99	6123.16	29
	H2B	3172.66	5065.12	4632.46	29
	H3A	3314.4	7237.08	6138.79	29
	H3B	3002.68	6361.48	7632.82	29
	H4A	4531.84	5785.94	6187.46	32
	H4B	4217.18	4839.93	7630.78	32
	H5A	4625.99	7984.91	7766.97	35
	H5B	4329.36	7017.03	9209.86	35
	H6A	5855.51	6533.98	7733.97	51
	H6B	5560.24	5608.37	9211.16	51
	H6C	5769.6	7376.26	9363.77	51

S2 Scanning Electron Micrographs of Hexyl Ammonium Lead Iodide Samples

The panels of Figure S1 compare the scanning electron microscopy (SEM) images of the hexyl ammonium lead iodide samples we synthesized for this study. As noted in our previous studies, the images in these panels so that the B samples formed under higher amine concentrations appear more granular and less plate-like. We hypothesized that this behavior results from early termination of the crystal growth process due to the presence of incompletely coordinated lead iodide polyhedra. In contrast, we find that the A samples formed under stoichiometric amine concentrations appear as well terminated crystalline plates. These images are consistent with the conclusion that we form fewer defects in these samples.

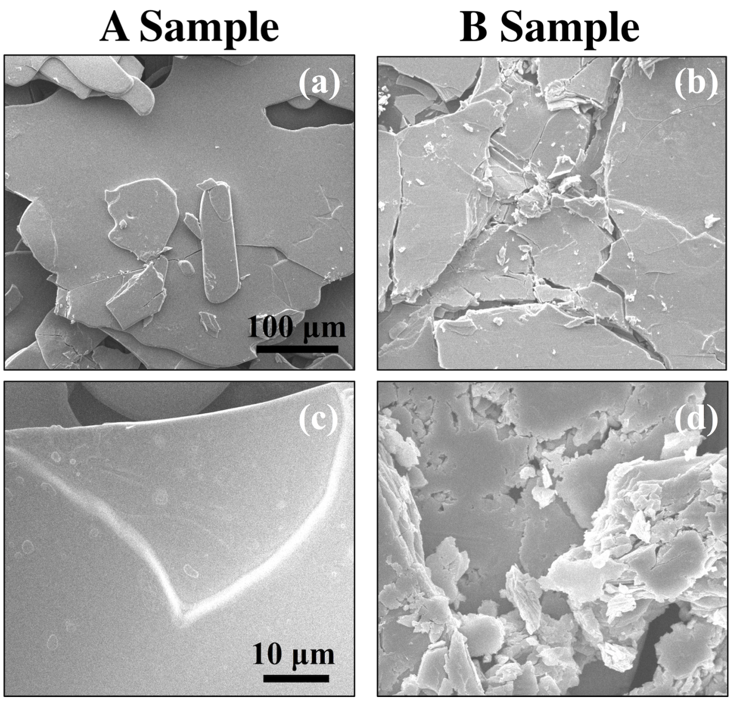

Figure S1: Comparisons of the SEM images of hexyl ammonium lead iodide A (panels a and c) and B (panels b and d) samples showing differences in their morphologies due to distinct defect concentrations.

S3 Analysis of Photoluminescence Spectra

We fit each PL spectrum of our HA_2PbI_4 samples to the following equation,

$$I_P(\omega) = A_1 e^{-[(\omega-\omega_1)/2\sigma_1]^2} + A_2 e^{-[(\omega-\omega_2)/2\sigma_2]^2} + \frac{A_3}{(\omega-\omega_3)^2+\Gamma_3^2} + A_4 e^{-[(\omega-\omega_4)/2\sigma_4]^2} \tag{S1}$$

$$+A_5 e^{-[(\omega-\omega_5)/2\sigma_5]^2} + A_6 e^{-[(\omega-\omega_6)/2\sigma_6]^2} + A_7 e^{-[(\omega-\omega_7)/2\sigma_7]^2} + A_8 e^{-[(\omega-\omega_8)/2\sigma_8]^2} + A_8 e^{-[(\omega-\omega_8)/2\sigma_8]^2},$$

where A_i and $\hbar\omega_i$ are the amplitude and resonant energy of the ith peak in the measured PL spectrum. For the X_3 feature we denote the width of the peak as Γ_3 while we use σ_i for the other i peaks, which are broadened inhomogeneously. The first four contributions to Eqn. (S1) correspond to the X_1, X_2, X_3, and X_4 features shown in Figure 1(c). The fifth, sixth, and seventh terms in Eqn. (S1) correspond to PL features in the X_D region showin in Figure 1(c). The eighth and ninth terms correspond to the features of the X_T region defined in Figure 1(c). Comparisons between experimental results and fits to the above model show that Eqn. (S1) can explain all the salient features of the experimental spectra.

The spectra that we measure at Spot 3 of the HA_2PbI_4 B Sample possess a distinct peak at 2321 meV that is not resolved by measurements at the other sample positions. However, as shown in Eqn. (S1), we need to include a feature in the models of our experimental near this energy to model all the other experimental results adequately. The appearance of this distinct peak in the B sample PL spectra at Spot 3 justifies the existence of a physical feature at this energy in the spectra we measure at all other spots.

S3.1 Comparison of Magneto-Photoluminescence Spectra of A and B Samples

Figure S2 compares the X_D and X_T regions of PL spectra we measured being emitted by our HA_2PbI_4 A and B samples under application of a 9 T magnetic field. This comparison shows that

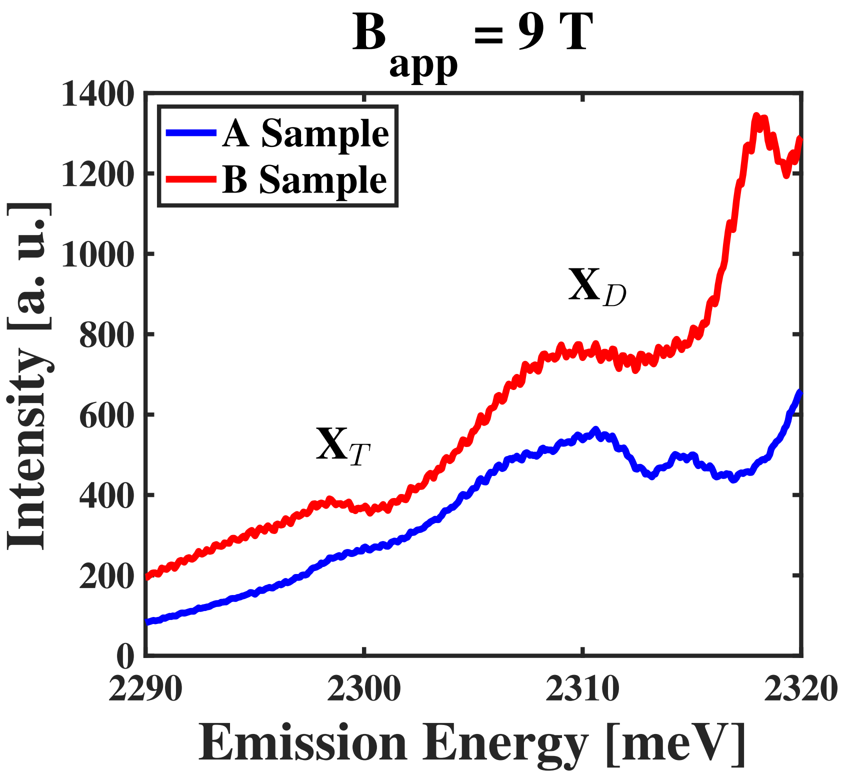

Figure S2: Comparison between the X_D and X_T regions of the A sample (blue) and B sample (red) PL spectra measured with a 9 T magnetic field applied in the Faraday configuration.

while we reduce the relative intensities of the X_D and X_T features in the PL spectra of the A sample, these features persist with appreciable intensities in the spectrum we measure being emitted by the B sample. This distinction correlates with the defect densities we expect to observe in the different samples based on the chemical conditions in which we form the materials. In the presence of more amine, as we used to make the B sample, there should be more defect sites formed, which would lead to larger PL intensities in the X_D region of the spectrum.

S3.2 Power Dependent-Photoluminescence Spectra

Figure S3 shows the PL spectra of the HA_2PbI_4 A sample for a range of incident laser powers. Each spectrum has been normalized to the intensity of the PL emission in the region around the X_3 and X_4 peaks. The comparison between the spectra shows that the intensity decreases in the X_D region while it increases in the X_T region. We use the data in this figure to construct the

power-dependent trends shown in Figure 2(b) of the main manuscript.

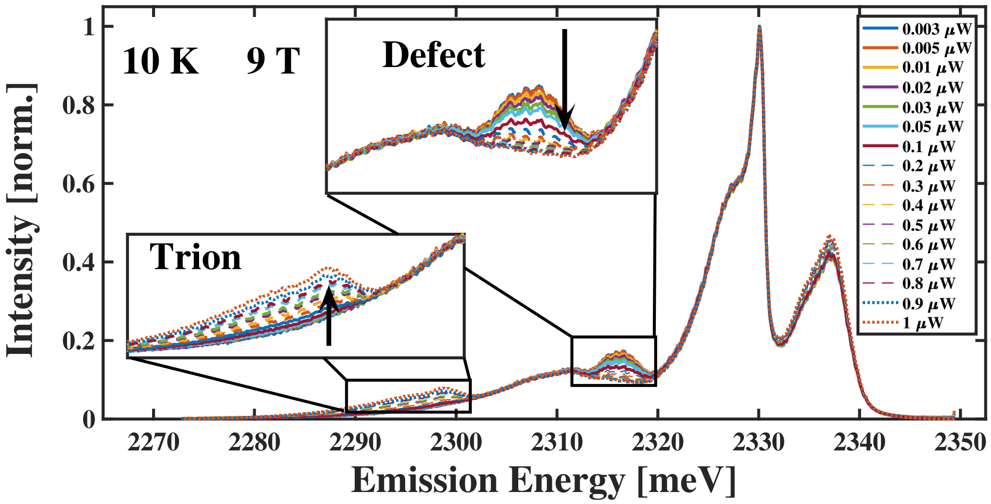

Figure S3: Incident laser power dependence of the normalized PL spectra we measure at spot 1 of the HA_2PbI_4 A sample. The insets highlight the behavior of the PL intensity in the regions of the X_D and X_T features.

The Figure 2(b) in the main manuscript shows the power dependence of the PL in the X_D and X_T spectral regions when we normalize the signal at every power to the peak near 2330 meV. We calculate the intensity at each point by integrating the raw signals between end points of each spectral region. We then fit the resulting trends to power law functions of the incident laser power, which we show in Figure 2(b).

Figure S4 shows the power dependence of the PL signal of the X_3 and X_4 extracted from fits using Eqn. (S1). Additionally, the insets in the panels of Figure S4 shows the dependence of the integrated intensities of the X_3 and X_4 features when plotted on a log-log scale. The slopes of these log-log trends are nearly unity, which implies that their intensity can be used to normalize all the power-dependent spectra effectively.

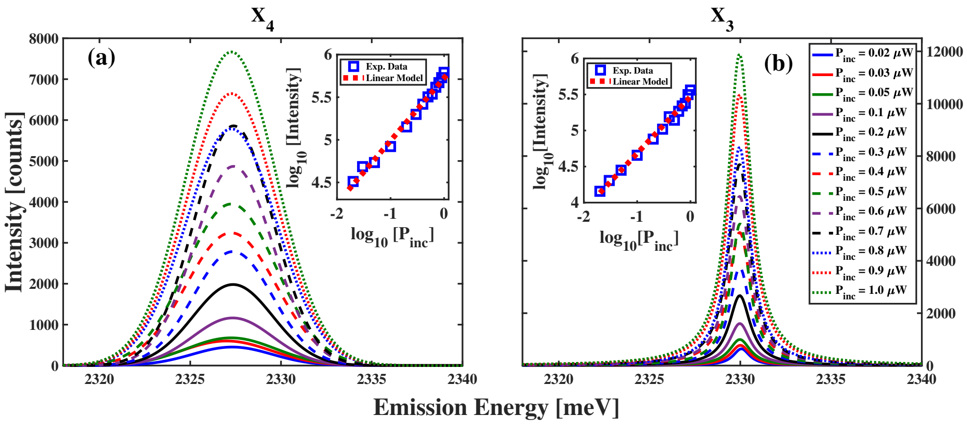

Figure S4: Incident laser power dependence of spectral features extracted from models of the HA_2PbI_4 A sample PL spectra corresponding to the X_4 exciton (a) and X_3 exciton (b) when measured at 10 K . Insets: comparison between the power dependence of the integrated intensity of the X_4 (a) and X_3 (b) features measured experimentally (blue squares) at 10 K to linear fits (dashed red) on the log-log scale.

S3.3 Spatial Inhomogeneity of X_D and X_T PL Features in HA_2PbI_4 B Sample

Figure S5 shows the variation in the relative intensities of features in the X_D and X_T regions of the B sample PL spectra. While we note these variations exist, we still observe the same features in each spectrum at generally the same energies. This reproducibility indicates that the PL spectra of this sample represents a universal material property, which we can use to understand the microscopic physics taking place following photoexcitation.

S3.4 X_{T_1} and X_{T_2} peaks in B Sample PL Spectra.

Figure S6 shows the X_{T_1} and X_{T_2} contributions to the model fit of the B sample PL spectrum we at spot 1 under 0 T at 10 K. As explained in the main manuscript, this figure demonstrates the difference in the widths of the peaks corresponding to each excitation. Additionally, we note that the energy and magnetic response of the X_{T_2} varies more significantly than that of X_{T_1}.

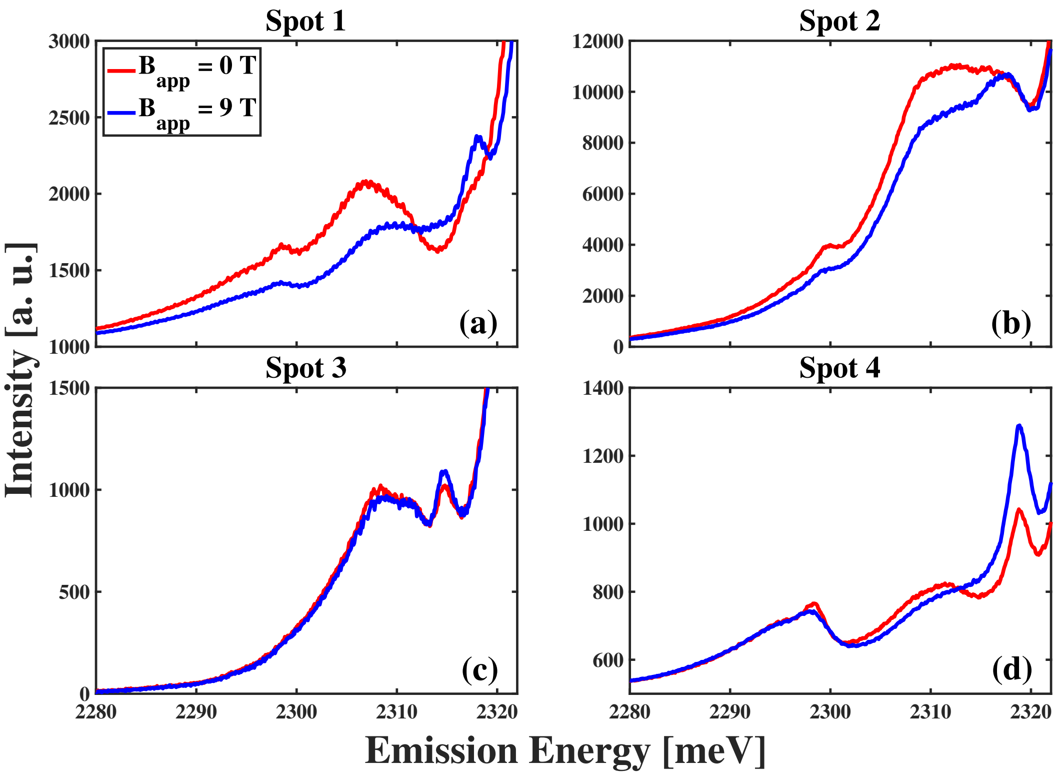

Figure S5: Comparison between the PL spectra of a HA_2PbI_4 B sample measured at 10 K with 0 T applied in a Faraday configuration (red) to that measured with 9 T applied in a Faraday configuration at Spot 3 while holding the sample temperature at 10 K (a) and 1.6 K (b). Magneto-PL difference spectra constructed by subtracting the spectrum measured at Spot 3 for 0 T applied magnetic field from that measured at 9 T while holding the HA_2PbI_4 B sample at 10 K (c) and 1.6 K (d).

S3.5 Variation of X_D g-factors

Figure S7 shows the variation in the magnetic response of the X_D peak around 2317 meV found in the PL spectra measured at different spots of the HA_2PbI_4 B sample.

S3.6 Temperature dependence of X_D features at Spot 3 of HA_2PbI_4 B sample

The panels of Figure S8 compare the magneto-PL spectra of our HA_2PbI_4 B sample measured at 10 K and 1.6 K when applying 9 T magnetic fields in opposite lab frame directions. While we find a low intensity peak at 2314 meV when holding the sample at 10 K as seen in Figure S8(a),

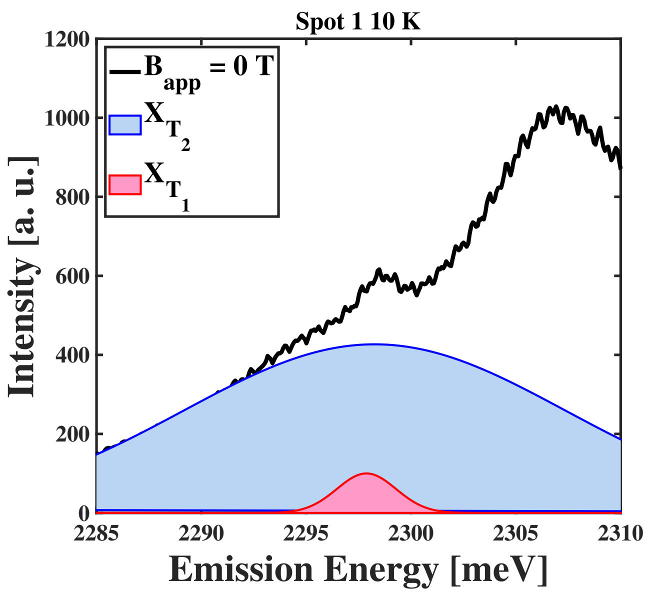

Figure S6: Comparison between the PL spectrum of our HA_2PbI_4 B sample measured 10 K under 0 T applied magnetic field (solid black) to X_{T_1} (filled red) and X_{T_2} (filled blue) contributions to models used to explain the experimental results. Features at higher energy than the X_{T_1} and X_{T_2}states lie in the X_D

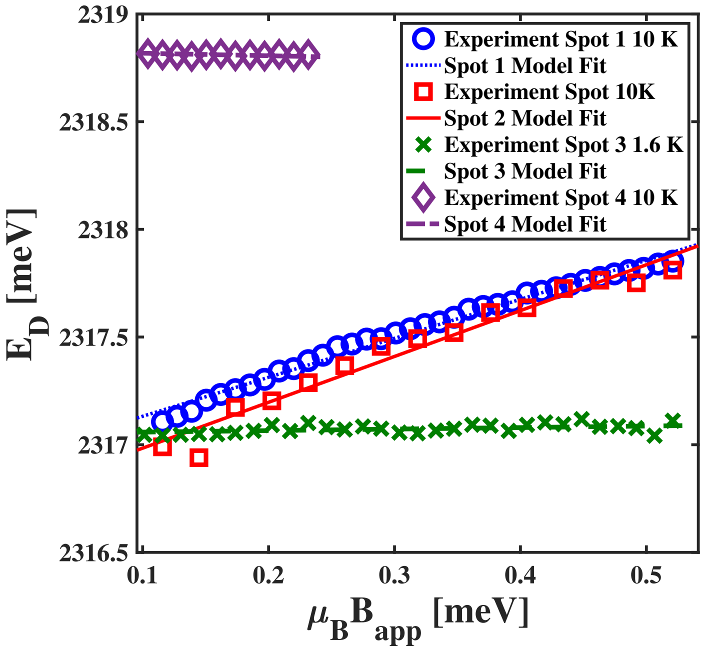

Figure S7: Comparisons of the experimental PL emission peak energy of the X_D exciton of the HA_2PbI_4 B sample as a function of applied magnetic field strength measured at 10 K (blue circles) to fits of these data to Eqn. (S2) (red line).

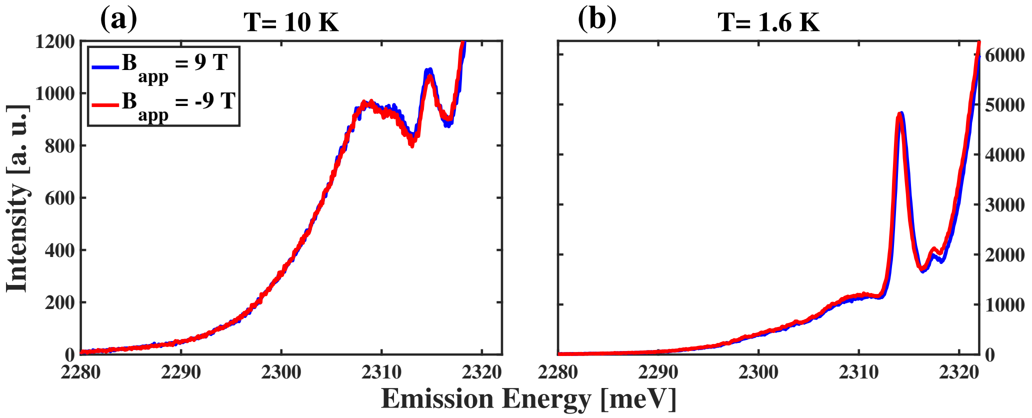

Figure S8: Comparison between the PL spectra of a HA_2PbI_4 B sample measured at Spot 3 with -9 T applied in a Faraday configuration (red) to that measured with +9 T applied at 10 K (a) and 1.6 K (b).

we find that the intensity of the PL feature at 2314 meV increases significantly upon cooling the sample to 1.6 K, which is observed in Figure S8(b). This behavior mimics what we observe at Spot 1 upon cooling the B sample to 2 K, as shown in Figure 2(d). Hence, we observe consistent behavior amongst the spectra that we measure at each spot as we cool the sample to nearly 2 K. Our comparing the PL spectra that we measure at spot 3 of our HA_2PbI_4 B Sample when applying +9 T and -9 T magnetic fields will only identify those peaks resulting from charged states that respond according to Zeeman coupling,

$$E_D(B_{app}) = E_D(0) + (g_e - g_h)\mu_B B_{app}, \tag{S2}$$

as considered in the Results and Discussion section of the main manuscript. This conclusion enables us to characterize changes in the sample's magnetic response at when we reduce the material temperature from 10 K to 1.6 K.

S3.7 Incorporating Spin-Orbit Coupling in Electronic Structure of Localized Defect States

As shown by several studies,[1–3] the layered nature and chemical compositions of HOIP SAQWs introduces a structural asymmetry and significant spin-orbit coupling, respectively, which leads to splitting these materials' conduction band (CB) and valence band (VB) along different reciprocal space directions in the material's Brillouin zone: the Rashba effect.[4,5] For the specific case of HOIP SAQWs formed from lead cations and halide anions, theory shows the VBs possess angular momentum from the electron spins alone while spin-orbit coupling of the 6p orbitals of Pb^{2+} cations to electron spin produces $J = \pm 1/2$ CBs.[6] The bands comprised of opposing total angular momenta cross at $k = 0$, which leads to the formation of Dirac points in both the VB and CB. Recent studies demonstrate the excitation of these materials at 2.54 eV in the absence of an applied magnetic field drives transitions between these Dirac points.[2]

To test if the Rashba effect changes the atomic orbital contributions to the defect states computationally, we extended our band structure calculations of the defective supercell defined above to include spin-orbit coupling, as shown in Figure S8. As demonstrated previously, these DFT calculations can estimate the Rashba splitting in HOIP SAQWs on the same order of magnitude as observed in experiments.[1–3] However, when we assess our results, we find that the identity of the defect state remains the unchanged relative to our calculations reported in the main manuscript.

S3.8 Spatial Variation in E_{T_2} peak energies in B Sample PL Spectra.

Figure S9 shows the variation in the response of E_{T_2} to an applied magnetic as a function of spot in our B Sample where we make the PL measurement. Interestingly, we find that this peak can respond in both linear and non-linear ways to the applied magnetic fields, which suggests that it's properties depend more significantly on environmental parameters than those of X_{T_1}. We fit the peak energies measured at spot 1 to a linear fit, which can explain the peak shifts adequately. We use a quadratic model to fit the peak energies we measure at spot 4, which necessitates implement-

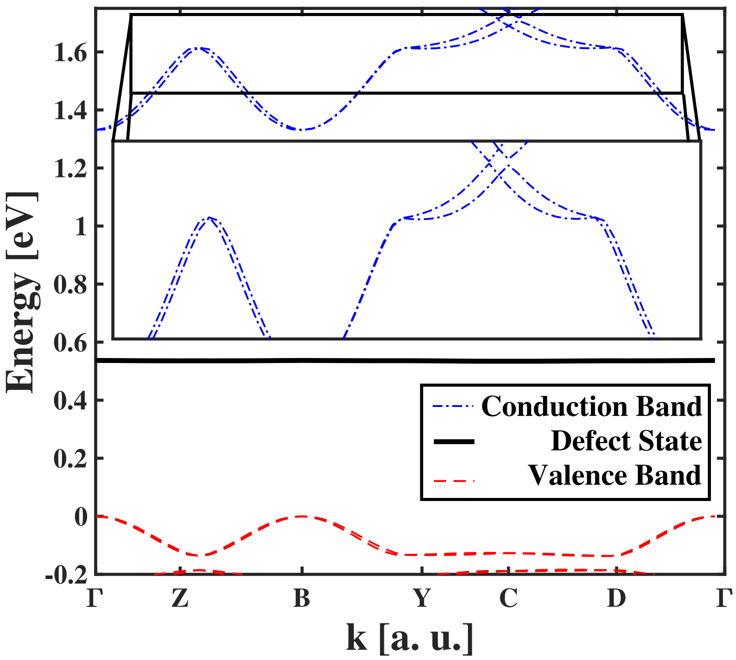

Figure S9: Electronic band structure of a defective HA_2PbI_4 supercell including the effects of spin-orbit coupling. The reader should note minimal changes in the characteristics of the defect state, which results from contributions by N p_z and I p_y atomic orbitals.

ing a negative quadratic coefficient to explain the experimental data adequately.

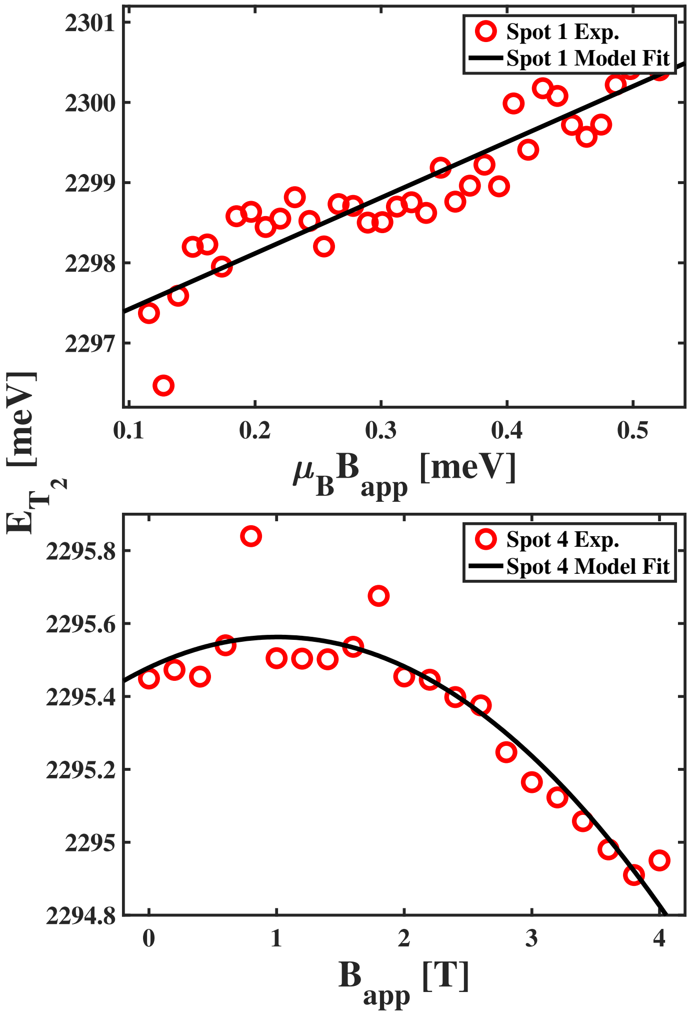

Figure S10: Comparisons of the experimental PL emission peak energy of the X_{T_2} exciton of the HA_2PbI_4 B sample at spot 1 (top) and spot 4 (bottom) as a function of applied magnetic field strength at 10 K (circles) to fits of these data to linear (top, solid line) and nonlinear models (bottom, solid line).

References

(1) Zhai, Y.; Baniya, S.; Zhang, C.; Li, J.; Haney, P.; Sheng, C.-X.; Ehrenfreund, E.; Vardeny, Z. V. Giant Rashba Splitting in 2D Organic-Inorganic Halide Perovskites Measured by Transient Spectroscopies. *Science Advances* **2017**, *3*, e1700704.

(2) Liu, X.; Chanana, A.; Huynh, U.; Xue, F.; Haney, P.; Blair, S.; Jiang, X.; Vardeny, Z. V. Circular photogalvanic spectroscopy of Rashba splitting in 2D hybrid organic–inorganic perovskite multiple quantum wells. *Nat. Comm.* **2020**, *11*, 323.

(3) Spencer, M. S.; Fu, Y.; Schlaus, A. P.; Hwang, D.; Dai, Y.; Smith, M. D.; Gamelin, D. R.; Zhu, X.-Y. Spin-orbit–coupled exciton-polariton condensates in lead halide perovskites. *Sci. Adv.* **2021**, *7*, eabj7667.

(4) Pfeffer, P.; Zawadzki, W. Spin splitting of conduction subbands in III-V heterostructures due to inversion asymmetry. *Phys. Rev. B - Cond. Matt. Mat. Phys.* **1999**, *59*, R5312–R5315.

(5) Trushin, M.; Výborný, K.; Moraczewski, P.; Kovalev, A. A.; Schliemann, J.; Jungwirth, T. Anisotropic magnetoresistance of spin-orbit coupled carriers scattered from polarized magnetic impurities. *Phys. Rev. B - Cond. Matt. Mat. Phys.* **2009**, *80*, 134405.

(6) Li, J.; Haney, P. M. Circular photogalvanic effect in organometal halide perovskite $CH_3NH_3PbI_3$. *Appl. Phys. Lett.* **2016**, *109*, 193903.
